# Ultrasound Beyond Joints: A Review of Extra-Articular Applications in Rheumatology

**DOI:** 10.1007/s11926-025-01186-9

**Published:** 2025-03-04

**Authors:** Emilio D’Ignazio, Davide Corradini, Tomas Cazenave, Riccardo Bixio, Caterina Baldi, Harjit Kaur Ubhi, Kate Smith, Richard J. Wakefield, Paul Emery, Andrea Di Matteo

**Affiliations:** 1 Department of Medicine, Surgery and Neurosciences, Rheumatology Unit, University of Siena, Siena, Italy; 2 Rheumatology Unit, Department of Medicine & Public Health, AOU and University of Cagliari, Cagliari, Italy; 3 Institute of Psychophysical Rehabilitation, Rheumatology Section, Buenos Aires, Argentina; 4 Rheumatology Unit, Department of Medicine, University of Verona, Verona, Italy; 5 NIHR Leeds Biomedical Research Centre, Leeds, UK; 6 Leeds Institute of Rheumatic and Musculoskeletal Medicine, University of Leeds, Leeds, UK; 7 Leeds Teaching Hospital NHS Trust, Leeds Institute of Rheumatic and Musculoskeletal Medicine (LIRMM), Chapel Allerton Hospital, Leeds, UK

**Keywords:** Ultrasound, Extra-articular, Interstitial lung disease, Large vessel vasculitis, Salivary gland, Muscle, Nerve, Skin, Nail

## Abstract

**Purpose of Review:**

This review highlights key ultrasound applications for evaluating extra-articular involvement in rheumatic diseases, including the lungs, vessels, salivary glands, muscles, nerves, skin, and nails. It explores recent advances, emerging areas of assessment, and future research directions. Additionally, the review examines current limitations in the routine use of ultrasound for these purposes and considers the potential of new technologies, such as shear-wave elastography, contrast-enhanced ultrasound, and artificial intelligence, to enhance the early detection and monitoring of extra-articular manifestations in rheumatic diseases.

**Recent Findings:**

Extra-articular manifestations in patients with rheumatic diseases are crucial for diagnosis, management (including treatment strategies), and prognosis, making accurate assessment essential. Growing evidence supports the role of ultrasound in assessing these manifestations for diagnosis, monitoring, and gaining insights into disease pathogenesis. Recent studies emphasize the significant utility of ultrasound in evaluating extra-articular involvement across various organ systems, including the lungs, vessels, salivary glands, muscles, nerves, skin, and nails. Technological advances, such as shear-wave elastography, contrast-enhanced ultrasound, and artificial intelligence, are expanding the scope and precision of ultrasound applications. Despite its potential, challenges such as operator dependency, lack of standardized protocols, and the need for specialized training hinder its widespread adoption.

**Summary:**

Ultrasound is a non-invasive, cost-effective, and radiation-free imaging modality with high diagnostic accuracy, making it a valuable tool for assessing extra-articular manifestations in rheumatic diseases. Emerging technologies may further enhance its clinical utility. However, efforts to standardize techniques and improve accessibility are necessary to optimize its integration into routine practice.

## Introduction

For many years, ultrasound has played a pivotal role in rheumatology, primarily focusing on the evaluation of musculoskeletal structures, including joints, tendons, and peri-articular soft tissues. Ultrasound allows clinicians to directly observe inflammatory changes in synovial joints, assess synovitis, tenosynovitis, and enthesitis, and detect structural damage, such as bone erosions [1]. As a highly accessible, non-invasive, and radiation-free imaging tool, ultrasound has proven invaluable for diagnosing, differentiating, and assessing disease activity in inflammatory arthritis, including rheumatoid arthritis (RA) and psoriatic arthritis (PsA) [2].

While the traditional applications of ultrasound in joint disease are well-established, there has been growing recognition of its potential beyond the musculoskeletal system (3–4). In several rheumatic conditions, such as connective tissue diseases (CTDs), extra-articular manifestations play a primary role in diagnosis, disease pathogenesis, and treatment choice. Similarly, in other traditionally ‘joint-centered’ conditions like RA, there is increasing evidence supporting the importance of extra-articular organ involvement, such as interstitial lung disease (ILD), prompting rheumatologists to adopt a more holistic approach rather than focusing solely on joint evaluation. Ultrasound has proven to be a significant tool as a first-line evaluation for many of these extra-articular manifestations, making it crucial for rheumatologists to identify (if they are operators) and interpret ultrasound findings in this area, and an increasingly essential skill in modern rheumatology.

This review provides an overview of the main pathological ultrasound findings associated with extra-articular manifestations of rheumatic diseases affecting the lungs, vessels, salivary glands, muscles, nerves, skin, subcutaneous tissue, and nails. Furthermore, we discuss recent updates and definitions from international working groups focused on standardizing ultrasound use in rheumatic diseases, such as the Outcome Measures in Rheumatology (OMERACT), and we explore the advantages and limitations of ultrasound, highlighting its role in current clinical practice. A summary of the main ultrasound changes and applications in the assessment of extra-articular manifestations is provided in Table 1. Lastly, we examine potential future innovations in the field, including novel targets for extra-articular ultrasound evaluation, emerging techniques [e.g. shear-wave elastography (SWE), use of very high-frequency probes], and artificial intelligence (AI) integration.

**Table 1:** Main applications of ultrasound in the assessment of extra-articular manifestations in rheumatic diseases

Organ	Pathology	Main sonographic alteration	Description of sonographic alterations	Main applications of ultrasound
Lungs	*ILD*	B-line	Vertical hyperechoic reverberation artifact originating from the pleural line, extending to the bottom of the screen without fading and moving in sync with lung sliding.	High sensitivity and negative predictive value for the detection of ILD. Promising use for diagnosis and monitoring of ILD.
		Pleural irregularities	Disruption of the pleural line’s regularity, appearing thicker, nodular, or linear.	
Vessels	*LVV*	Halo sign	Homogenous, hypoechoic wall thickening, well delineated towards the luminal side, visible both in longitudinal and transverse planes, most commonly concentric in transverse scans.	Sensitive and specific for the diagnosis of LVV. The halo sign is included in the latest classification criteria for GCA.
		Compression sign	The thickened arterial wall remains visible upon compression; the hypoechogenic vasculitic vessel wall thickening contrasts with the mid-echogenic to hyperechogenic surrounding tissue.	
		Slope sign	Thickened segment of the inflamed arterial wall that transitions to a normal intima-media structure.	
Salivary glands	*SjD (and others)*	Gland parenchymal abnormalities	Main features include glandular echogenicity changes and irregular areas of hypoechoic and anechoic regions.	Promising role for diagnosing salivary involvement. Currently not included in the latest classification criteria for SjD. Potential use for identification of lymphoma
		Vascularization in gland parenchyma	Increased Doppler signal in gland parenchyma.	
Muscles	*IIM*	Change in muscle tissue echogenicity	In the early stages of myositis, some studies describe the muscle as hypoechoic, while others describe it as hyperechoic without loss of the underlying bone signal (“shine-through” or “see-through appearance”), with or without increased Doppler signal.	Promising role for diagnosis and monitoring of muscle involvement. To date, other imaging techniques, such as MRI (for IIM) and DXA (for sarcopenia) are recommended as first line imaging tools
	*Sarcopenia*		Decreased muscle mass (i.e. atrophy) Increased muscle echogenicity due to fat or fibrotic tissue replacement of muscle (i.e. loss of muscle quality)	
Nerves	*Compressive neuropathy (e.g. CTS)*	Nerve thickening	Increased CSA of the nerve.	Reference imaging tool for the detection of compressive neuropathy in accessible nerves. Useful for the evaluation of secondary causes of CTS (e.g. tenosynovitis of flexor tendons) Useful for guiding injections.
		Vascularization in the nerve	Increased Doppler signal within the nerve.	
Skin, sub-cutaneous tissues, and nails	*Scleroderma*	Skin thickness changes	Edematous phase: hypodermal thickening. Fibrotic phase: dermal and hypodermal thickening. Atrophic phase: dermis and hypodermis thinning.	Promising tool for assessing skin and sub-cutaneous soft tissue involvement, understanding disease pathogenesis, and defining treatment response in SSc patients. Mainly used in research settings.
	*Calcinosis cutis*	Sub-cutaneous calcifications	Well-defined hyperechoic deposits in the skin and subcutaneous tissues with or without posterior acoustic shadowing and/or surrounding Doppler signal	
	*Skin psoriasis*	Increased skin thickness	Thickening of epidermis and dermis, with a “four-band layout”. Acoustic shadowing and increased Doppler signal also seen in the upper dermis.	Promising tool for diagnosing and monitoring response to therapy in patients with psoriasis. Mainly used in research settings.
	*Nail psoriasis*	Increased nail thickness	Loss of trilaminar structure, with increased nail bed thickness and nail plate thickness. Often increased Doppler signal changes in the nail bed	

Legend. *CSA* = cross-sectional area; *CTS* = carpal tunnel syndrome; *DXA* = Dual-Energy X-ray Absorptiometry; *GCA* = giant cell arteritis; *IIM* = idiopathic inflammatory myopathies; *ILD* = interstitial lung disease; *LVV* = large vessel vasculitis; *MRI* = magnetic resonance imaging; *SSc* = systemic sclerosis; *SjD* = Sjögren disease

## Lungs

ILD is a frequent manifestation of CTDs, affecting up to 50% of systemic sclerosis (SSc), 20–60% of mixed connective tissue disease (MCTD), and 78% of idiopathic inflammatory myositis (IIM) patients [5]. ILD is also a common extra-articular manifestation of RA, where it is a major cause of morbidity and mortality [6].

Diagnosing ILD involves clinical, functional, and imaging assessments, though each method has limitations. Respiratory symptoms can be nonspecific, and appear late in the diseases. Pulmonary function tests (PFTs), while useful for monitoring, lack sensitivity in early or mild ILD [7]. Imaging, particularly chest x-rays, is less sensitive for early detection, while high resolution computed tomography (HRCT), which represent the imaging gold standard, is expensive and exposes patients to ionizing radiation [8].

**Fig. 1:**
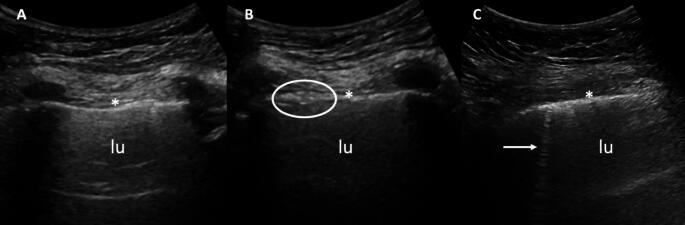
Ultrasound in lung involvement (interstitial lung disease). **A** shows a lung ultrasound using a convex probe, demonstrating a normal pleural line. **B** highlights pleural line irregularities, visible as nodular thickening (white circle), and **C** reveals a B-line (indicated by the white arrow) in patients with rheumatoid arthritis. *Asterisks* = pleural line; *LU* = lung parenchyma

Lung ultrasound (LUS) has emerged as valuable tool for assessing ILD in rheumatic diseases, particularly SSc but also other conditions, such as RA and Sjögren disease (SjD) [9, 10]. Key LUS features of ILD include B-lines and pleural irregularities, as reviewed by the OMERACT group [11]. Recently, OMERACT conducted a Delphi exercise to define consensus-based LUS findings in ILD, using SSc as a model [12]. B-lines were defined as “vertical hyperechoic reverberation artefacts originating from the pleural line, extending to the bottom of the screen without fading, and moving in sync with lung sliding”, representing subpleural interlobar septa occupation (oedema or collagen deposition); and pleural irregularity was described as “any disruption of the pleural line’s regularity, appearing thicker, nodular, or linear”. Examples of LUS findings indicating ILD (i.e pleural irregularity and B-line) have been illustrated in Fig. 1.

Several studies have highlighted the diagnostic value of LUS for ILD, including in patients with early SSc, even without respiratory symptoms, and RA [13–15]. A meta-analysis of 11 studies comparing LUS with HRCT in 487 patients with CTDs and RA found that the sensitivity and specificity of LUS were 0.982 (95% CI: 0.904–1.000) and 0.875 (95% CI: 0.710–0.965), respectively [16]. Defining a positive LUS for ILD as the presence of either B-lines or pleural irregularities increases diagnostic sensitivity for ILD to 100%, though it reduces specificity [17].

Studies indicate that LUS-measured pleural thickness, particularly between 3 and 5 mm, correlates with reticular-nodular patterns on HRCT, while thicknesses ≥ 5 mm are associated with the honeycomb pattern, suggesting a potential link between pleural thickness and fibrosis severity [18].

Beyond diagnostics, LUS shows promise as a prognostic tool for ILD in CTDs. An analysis of SSc patients found that basal B-lines significantly correlated with reduced diffusing capacity and forced vital capacity over a 12-month period [19]. A larger longitudinal study found that LUS B-lines predicted the onset of new ILD in SSc patients and disease progression in those with pre-existing ILD [20]. A recent study investigated the ability of LUS to monitor ILD progression in SSc patients receiving anti-fibrotic therapy; this study showed a consistent decrease in the total number of B-lines and pleural irregularities during treatment [21].

Despite its potential, LUS faces challenges in assessing ILD. One issue is the lack of consensus on the optimal number of sites to assess, with studies varying between 10 and 72 sites per patient. One study found a strong correlation between comprehensive (58 scanning sites) and simplified (14 scanning sites) assessments and HRCT findings, showing that a simplified approach can be as effective as an extended one due to the shorter time required for the examination [17]. B-line quantification also lacks standardization, with no clear consensus on whether quantitative (including the threshold of B-lines required to consider a patient positive for ILD), semi-quantitative, or dichotomous scoring methods are superior [22]. In a study conducted in 2018, a cut-off of 10 B-lines demonstrated the highest positive likelihood ratio (12.5) for identifying significant SSc-ILD [23]. Probe selection also varies, with convex probes often preferred for detecting B-lines, while linear probes, with their higher frequency, may be more suitable for identifying pleural irregularities [24, 35]. Multiple studies have reported high inter- and intra-rater reliability of LUS in evaluating ILD [15, 25, 26]. Conversely, a recent OMERACT study found only moderate inter-rater agreement among experts [11]. Additionally, B-lines can appear in various other pathological lung conditions, such as pulmonary edema, infection, chronic obstructive pulmonary disease, chronic heart failure, and acute respiratory distress syndrome, as well as in healthy individuals, which complicates ILD diagnosis [27–29]. Some studies have highlighted that, while the sensitivity of LUS and negative predictive value are high, its specificity might be suboptimal [16].

Regarding recent innovations of LUS is ILD, few studies have explored SWE for measuring pleural stiffness in ILD, with conflicting results [30]. Some studies reported increased SWE lung stiffness in ILD patients, but evidence remains scarce [31, 32]. AI and deep learning have also been evaluated in the context of ILD, particularly for diagnosis and subtype classification. A recent study applied these technologies to HRCT scan images and demonstrated that, in some cases, the model outperformed experienced radiologists [33]. To date, the application of AI in LUS for assessing ILD remains unexplored.

## Vessels

Vasculitis refers to a heterogeneous group of inflammatory diseases involving the blood vessels. The current classification categorizes vasculitis based on the size of the affected vessels (i.e., small, medium, or large-vessel vasculitis) [34]. To date, ultrasound has proven mainly useful in large vessel vasculitis (LVV), with giant cell arteritis (GCA) and Takayasu arteritis (TAK) being the most common types [35]. GCA typically affects adults over 50, particularly targeting the extracranial branches of the carotid artery and the axillar artery, while TAK primarily affects younger women targeting the aorta and its major branches [35]. Early diagnosis is crucial to prevent serious complications, such as vision loss in GCA and severe vascular damage (i.e. stenosis, occlusion, aneurism) in TAK [36–38].

Traditionally, temporal artery biopsy (TAB) has been the gold standard for diagnosing GCA, while angiography was the preferred method for TAK. Although TAB remains valuable, imaging techniques have gained prominence, as reflected in the latest European League Against Rheumatism (EULAR) recommendations [39–41]. These guidelines now suggest ultrasound as the first-line diagnostic tool, particularly for GCA. In addition, the ‘halo sign’ on temporal arteries has been included in the latest American College of Rheumatology (ACR)/EULAR classification criteria for GCA [39]. For TAK, EULAR recommends magnetic resonance imaging (MRI) as the preferred imaging modality because it provides comprehensive visualization of the aorta, offers better accuracy than angiography, and avoids the radiation exposure associated with positron emission tomography (PET)-computed tomography (CT). However, ultrasound remains useful for evaluating peripheral arteries in TAK, especially when MRI is not readily available [41].

**Fig. 2:**
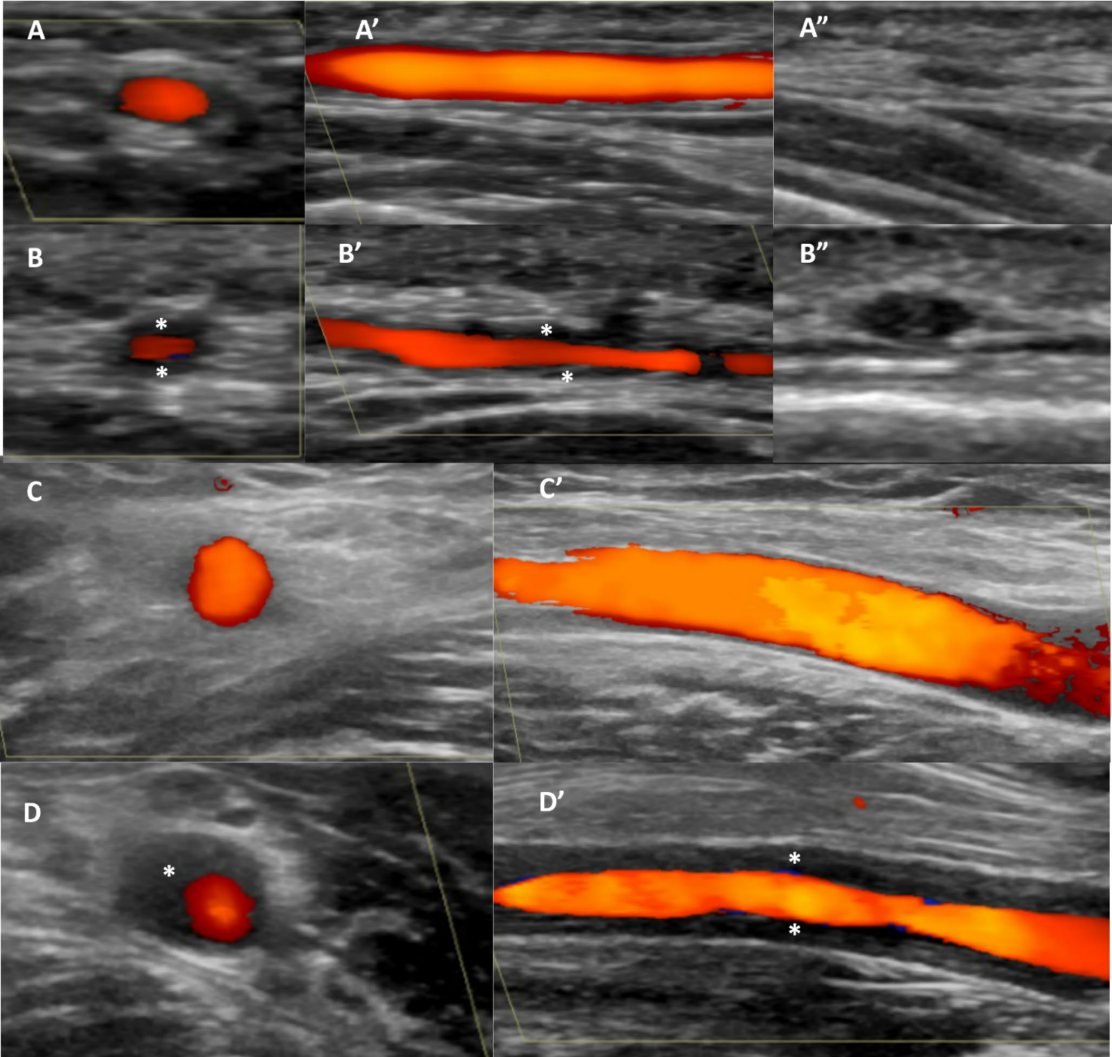
Ultrasound in the assessment of vessels (large vessel vasculitis). **A** shows the ultrasound scan of the superficial temporal artery in a healthy subject, demonstrating normal vessel wall thickness in both transverse (**A**) and longitudinal (**A’**) views. The vessel wall disappears upon probe compression (**A’’**). The figure also includes images of the superficial temporal artery in a patient with giant cell arteritis, displaying the halo sign in transverse (**B**) and longitudinal (**B’**) views (marked with asterisks). In **B’’**, the thickened arterial wall remains visible despite external compression, indicating the compression sign. **C** presents a normal axillary artery in transverse (**C**) and longitudinal (**C’**) views in a healthy subject. **D** and **D’** display the halo sign in the axillary artery in transverse and longitudinal views, respectively, in a patient with Takayasu disease

The OMERACT working group identified two key ultrasound signs for LVV: the halo sign and the compression sign [42]. Originally introduced by Schmidt et al. [43], the halo sign was later defined by OMERACT as “homogenous, hypoechoic wall thickening, well delineated towards the luminal side, visible both in longitudinal and transverse planes, most commonly concentric in transverse scans” [42]. The compression sign occurs when the thickened arterial wall remains visible despite external compression, unlike surrounding tissue [42] (Fig. 2). Other ultrasound signs of vessel inflammation have also been described, such as the slope sign, which reflects the transition between thickened, inflamed arterial walls and normal intima-media structures [44]. The intima-media thickness (IMT) is another important feature detectable by ultrasound and is related to the halo appearance; cut-off values for normal IMT have been proposed for different arteries: in GCA, the common superficial temporal artery shows values of 0.42 mm, while the frontal branch has a cut-off of 0.34 mm, the parietal branch 0.29 mm and the axillary artery 1.0 mm [45]. Traditionally, the temporal artery (including the superficial temporal artery, parietal and frontal) and the axillary artery have been the sites most commonly studied via ultrasound for the diagnosis of LVV. However, virtually any artery can be affected in these conditions, with more extensive protocols sometimes including the subclavian, vertebral, brachiocephalic, and carotid arteries [46, 47].

Regarding the diagnostic accuracy of ultrasound in LVV, a meta-analysis comparing imaging modalities for GCA found that ultrasound had superior sensitivity (88%) compared to MRI (81%) and PET-CT (76%), though all three methods showed high specificity (above 96%) [48]. PET-CT was particularly useful for detecting axillary artery involvement, while ultrasound was more sensitive for temporal arteries [49]. Studies have also reported strong agreement between ultrasound and PET-CT, although regional discrepancies in different arteries persist [50, 51]. Additionally, MRI shows high concordance with ultrasound findings in GCA [52]. In the TABUL study, the authors compared sensitivity, specificity and cost-effectiveness of ultrasound compared with biopsy or ultrasound combined with biopsy for diagnosing GCA. Interestingly, the combination of clinical judgment with biopsy demonstrated a sensitivity of 91% and specificity of 81%, while the combination with ultrasound showed higher sensitivity (93%) but slightly lower specificity (77%) [53]. Incorporating axillary artery assessment into ultrasound protocols, in addition to temporal artery evaluation, increases sensitivity to 89% while maintaining specificity above 90% [54].

A few studies have showed the promising role of ultrasound in the monitoring of LVV [55, 56]. Ultrasound monitoring can be particularly useful in cases where elevated inflammatory markers accompany non-specific symptoms or when a new ischemic event or worsening of disease-related ischemia occurs. Imaging is also valuable for patients treated with interleukin-6 pathway inhibitors, as inflammatory markers are not clinically informative in these situations [49].

An important area of research involves the development of scoring systems to enhance the diagnostic and monitoring capabilities of ultrasound for LVV. The “Halo Count” (HC) and “Halo Score” (HS), which measure the number and thickness of halos, respectively, have shown excellent diagnostic accuracy with areas under the curve of 0.892 for HC and 0.921 for HS [57]. Other recent scoring systems include Southend Halo Score and OMERACT GCA ultrasound score (OGUS) [58, 59]. Further studies are needed to confirm the value of these scoring systems in the monitoring of the disease.

Furthermore, ultrasound has been shown to detect subclinical GCA in 22% of patients with polymyalgia rheumatica. Whether these patients with subclinical vessel inflammation (and potentially more severe phenotypes with an increased rate of relapse) should be treated as if they have GCA is a topic of ongoing discussion and research [60].

A potential challenge in interpreting ultrasound findings for vascular inflammation is the use of glucocorticoids, which remain a cornerstone in the treatment of LVV because their use could mask vessel inflammation signs. EULAR recommends immediate treatment with glucocorticoids for patients suspected of having GCA to prevent vision loss and other ischemic complications [61]. Imaging (including ultrasound) should be performed within 72 h of treatment initiation, as this period does not significantly affect diagnostic accuracy [62, 63].

Another concern is validating IMT cutoffs for patients with significant arteriosclerosis, as cardiovascular risk factors can influence IMT measurements (i.e. diabetes and hypertension), particularly in older individuals [64, 65]. Finally, other conditions, such as granulomatosis with polyangiitis or eosinophilic granulomatosis, which can affect the temporal arteries or cause periaortitis, may mimic the ultrasound appearance of GCA and should be considered in the differential diagnosis [66].

Technological advancements such as high-frequency probes (50–70 MHz) offer superior resolution for distinguishing arterial layers, though these devices are costly and less suited for musculoskeletal imaging being limited by lower depth of penetration [67]. Additionally, contrast-enhanced ultrasound holds promise for detecting increased wall perfusion in active GCA and TAK, particularly in the common carotid arteries [68]. Finally, AI has been tested for diagnosing GCA. This tool achieved high specificity (95%) but a moderate sensitivity (60%), with images acquisition identified as the main limitation, despite these were obtained by expert operators [69].

## Salivary Glands

Salivary glands are target organs in various rheumatic conditions, particularly SjD, but they are also affected by other autoimmune diseases, including systemic lupus erythematosus (SLE), SSc and RA [70]. SjD leads to a progressive decline in the function and structure of the salivary glands due to chronic inflammation and immune-mediated damage. This deterioration results in significant clinical symptoms such as xerostomia, xerophthalmia, and potential complications, including an increased risk of lymphoproliferative disorders if not managed properly [70].

Diagnostically, the major salivary glands, such as the parotid, submandibular, and sublingual, are significant in autoimmune rheumatic diseases like SjD due to their larger size and accessibility for imaging, particularly through ultrasound, which facilitates the detection and assessment of parenchymal changes associated with these conditions [71].

The use of ultrasound to evaluate salivary glands in SjD was first proposed over 30 years ago, and interest has grown among rheumatologists and radiologists [72, 73]. A recent systematic review identified a large number of ultrasound scoring systems for assessing salivary glands in SjD patients [74]. Ultrasound has shown high diagnostic accuracy, with one systematic review reporting a pooled sensitivity of 80% and specificity of 90% for diagnosing primary SjD [75]. Correlations between ultrasound findings and traditional diagnostic methods—such as histology (the traditional gold standard), MRI, and salivary flow measurements—have established ultrasound as a reliable diagnostic tool. Moreover, a negative ultrasound scan in suspected SjD patients has demonstrated high negative predictive value in multiple studies, thereby potentially reducing the need for unnecessary biopsies [76].

**Fig. 3:**
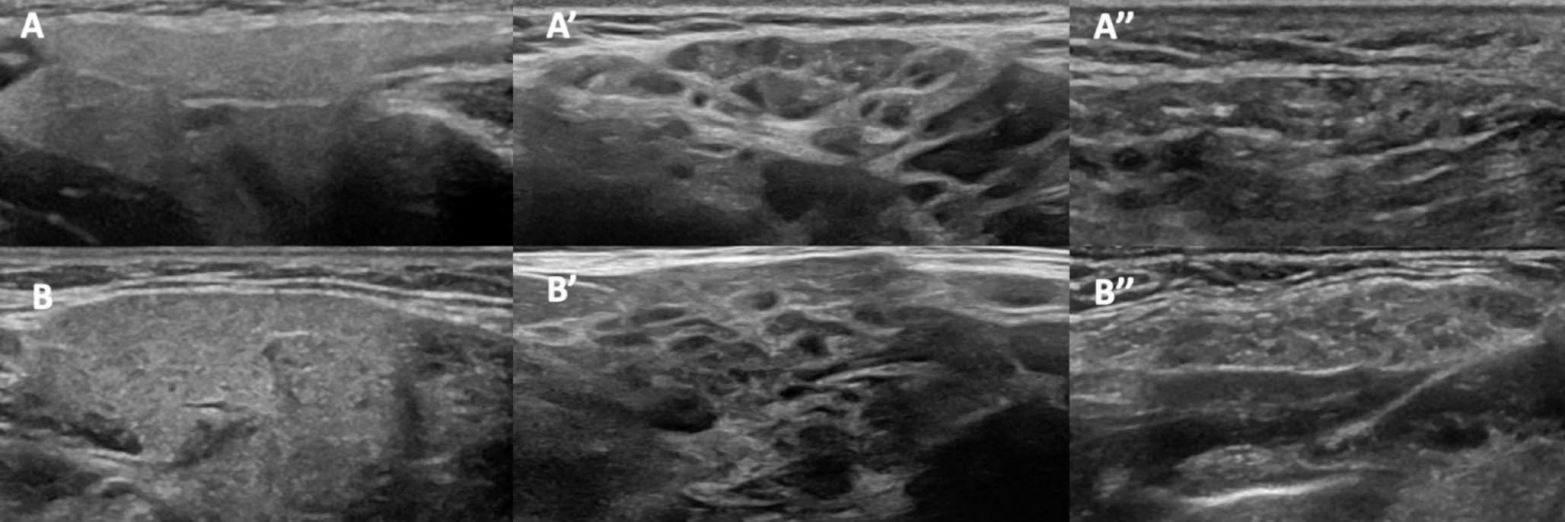
Ultrasound in the assessment of salivary glands. **A** and **B** present ultrasonographic images of the parotid and submandibular glands, respectively, showing the homogeneous structure of gland parenchyma in a healthy subject. **A’** and **B’** depict grade 3 inhomogeneity in patients with Sjögren disease, characterized by anechoic and hypoechoic areas affecting the entire gland surface (parotid and submandibular gland, respectively). **A’’** and **B’’** show diffuse atrophy and a loss of clear demarcation between the gland border and surrounding tissue in other patients with Sjögren disease (parotid and submandibular gland, respectively)

In 2019, the OMERACT ultrasound group developed a semi-quantitative scoring system (ranging from 0 to 3) to assess salivary glands in primary SjD, focusing on the parotid and submandibular glands while excluding the sublingual glands due to their small size [77]. This system evaluates each gland based on two key pathological features: the degree of inhomogeneity of the glandular echogenicity and the presence of hypoechoic or anechoic areas within the parenchymal tissue (Fig. 3). A score of 2 or higher in at least one gland is conventionally considered pathological [78]. Prior to this, the OMERACT group conducted a reliability exercise to standardize core definitions of salivary gland abnormalities, assessing features beyond echogenicity and homogeneity, such as the presence of hyperechoic bands (which may indicate fibrosis), the number of abnormal lymph nodes within the glands, calcifications, the visibility of the posterior border of the glands, and the visibility of normal lymph nodes at the poles of the parotid glands [79]. Among these, echogenicity and homogeneity emerged as the most reliable ultrasound findings for identifying salivary gland abnormalities [79]. Subsequently, the OMERACT group developed another scoring system utilizing Doppler signal to assess glandular inflammation, where a higher Doppler signal corresponds to greater inflammation. However, physiological blood flow and external stimuli (e.g. food intake, stress) can influence Doppler signal, which need to be considered when interpreting the findings [80].

Ultrasound of salivary glands has also showed potential for monitoring disease progression in SjD. Trials involving rituximab have demonstrated reductions in salivary gland echogenicity in treated patients [81]. However, since no treatment has been definitively proven to halt or reverse disease progression, the role of ultrasound in monitoring remains to be fully defined. Notably, ultrasound has shown value in detecting lymphoproliferative diseases, a serious complication of SjD, with ultrasound-guided biopsies increasingly being used to diagnose suspected malignancies [82]. Similarly, there is also growing interest in whether ultrasound-guided biopsies could replace traditional labial biopsies for diagnosing SjD [83].

Despite the promising use of ultrasound in assessing salivary glands in SjD and other rheumatic conditions, such as RA and SLE, several challenges limit its routine clinical application. Notably, ultrasound is not included in the 2016 ACR/EULAR classification criteria for primary SjD [84]. The use of Doppler to assess glandular inflammation is promising, but further research is needed to clarify its diagnostic value and sensitivity to change. When the OMERACT grey scale scoring system was tested in patients, the reliability for the submandibular gland was suboptimal (kappa 0.44), while moderate reliability (kappa 0.62) was observed for both glands in an inter-reader reliability exercise [85].

Emerging technologies, such as SWE and AI, may provide additional tools for more accurate diagnosis and monitoring of disease progression. SWE could help detect early fibrotic changes, while AI may assist in standardizing image interpretation and scoring [86, 87]. Further research is expected to focus on the integration of these advanced imaging techniques into routine scanning protocols.

## Muscle

### Inflammatory Idiopathic Myopathy

IIM, collectively known as myositis, encompasses a group of heterogeneous disorders characterized by muscle weakness and inflammation [88]. While symptoms, clinical examinations, and serological assessments are essential for evaluating patients with suspected IIM, additional instrumental examinations may be required for a definitive diagnosis. Muscle biopsy remains the gold standard; however, it is an invasive procedure and may not always be feasible. Electromyography can help differentiate between myogenic and neurogenic disorders, but it can often yield nonspecific results, especially in earlier phases and its interpretation is operator-dependent [89].

In recent years, there has been increasing interest in the role of imaging for IIM, although no classification or diagnostic criteria currently incorporate imaging methods [90, 91]. MRI is the reference imaging method for IIM, providing the best accuracy for detecting muscle oedema, fatty replacement, and muscle atrophy—key features of these conditions. However, MRI has practical limitations, including high costs, limited availability, and its usual restriction to evaluating a single muscle area at a time [92].

Ultrasound has the ability to evaluate different aspects of muscle involvement, mainly muscle mass and muscle quality [93]. Compared to MRI, ultrasound offers greater patient acceptability and allows for dynamic and multi-site assessment.

**Fig. 4:**
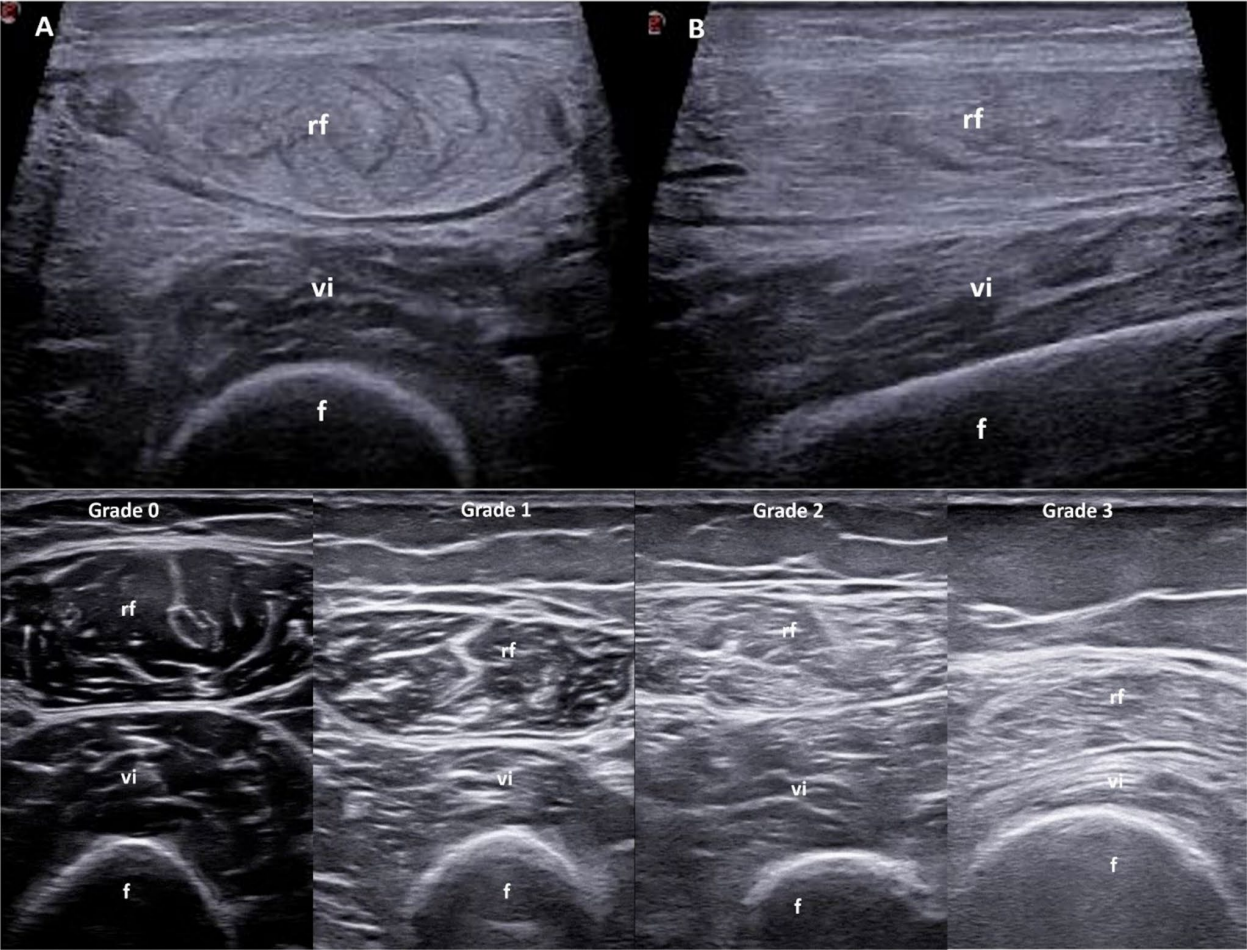
Ultrasound in muscle involvement (inflammatory myositis and sarcopenia). **A** shows a transverse scan, and **B** a longitudinal scan, both demonstrating widespread increased echogenicity without loss of the underlying bone signal (“see through” appearence) of the rectus femoris muscle with preserved muscle structure in a patient with polymyositis. Additionally, an example of a recently developed semi-quantitative scale for assessing muscle echogenicity (quadriceps muscle) in patients with sarcopenia is provided (reference no. 112). Images were obtained from patients with rheumatoid arthritis. *f* = femur; *rf* = rectus femoris muscle; *vi* = vastus intermedius muscle

One of the earliest studies investigating the correlation between histopathology and muscle ultrasound in IIM found that affected muscle areas with oedema appeared hypoechoic and thicker than unaffected areas [94]. In a recent study comparing ultrasound and MRI in patients with IIM, grey scale oedema on ultrasound was described as an area of decreased echogenicity (hypoechogenicity) [95]. However, there are descriptions in the literature where ultrasound in the acute phase may show muscle edema as a generalized increase in echogenicity (hyperechoic) without loss of the underlying bone signal (“shine-through” or “see-through” appearance) [93]. This highlights uncertainties in the appearance of muscle on ultrasound in the acute phase, with reports of both hypoechogenicity and hyperechogenicity (Fig. 4). This discordance is partly due to the limited number of histological studies recently analyzing correlations in patients with this condition. Ongoing research is focused on further clarifying the ultrasound appearance of patients with IIM, including the different phases of the disease (acute, chronic, atrophic), as highlighted by recent OMERACT studies [96].

Muscle thickness appears normal or mildly increased in the acute phases and decreases over time [97]. Some authors have suggested evaluating fascial thickness and perimysial septa, but the data remain limited and the definitions uncertain [98]. While Doppler signals have shown promise in identifying active inflammation [99], further clarification is needed to establish their reliability in diagnosing IIM and assessing treatment response.

New promising tools are emerging in muscle assessment, but few data are currently available. SWE, an advanced ultrasound technique providing quantitative measurements of muscle elasticity/stiffness, has yielded contrasting results; indeed, some studies report a reduction in muscle stiffness, while others have shown inflamed muscles exhibiting greater stiffness in patients with IIM [100, 101]. Contrast-enhanced ultrasound has been shown to correlate with MRI oedema in patients with histologically defined myositis [102]. Recent studies have also focused on the application of AI and deep learning algorithms, which can assist in the earlier and more accurate classification of myositis using ultrasound [103]. However, data on the use of these tools remain limited.

### Sarcopenia

Sarcopenia, as defined by the European Working Group on Sarcopenia in Older People 2 (EWGSOP2), is a progressive disorder characterized by the loss of skeletal muscle mass and strength, which increases the risk of adverse outcomes such as falls, fractures, physical disability, and mortality [104, 105]. Sarcopenia is traditionally classified into primary sarcopenia, which is age-related, and secondary sarcopenia, which arises from other conditions, including rheumatic diseases [104].

Imaging is crucial for evaluating muscle mass and quality, which are main criteria for the diagnosis of sarcopenia [106]. Although dual-energy X-ray absorptiometry (DXA), CT, and MRI are considered gold standards, their use is often limited by availability, costs, and concerns about radiation exposure [107].

In patients with sarcopenia, ultrasound can detect both quantitative changes, such as muscle atrophy (i.e. reduced muscle thickness), and qualitative changes, like increased echogenicity, which may indicate fibrosis or fat infiltration of muscle [108].

While ultrasound can evaluate multiple muscle groups, the quadriceps muscle is frequently selected in both clinical practice and research due to its established reliability and correlation with reference imaging methods, like MRI and CT [109, 110].

Several techniques have been developed to assess muscle echogenicity using ultrasound. The Heckmatt score, a semi-quantitative four-grade scale introduced in 1982 for pediatric neuromuscular patients [111], was recently modified by Di Matteo et al. to create a new semi-quantitative scale for evaluating muscle echogenicity in patients with rheumatic diseases [112–114]. An example of this newly proposed scale is shown in Fig. 4. Another semi-quantitative scale, developed by Möller and colleagues, uses a three-point system (normal, moderate, severe) to assess muscle echogenicity [115]. This scale, based on cadaveric histological data, has demonstrated high inter- and intra-rater reliability.

Ultrasound has the potential to serve as a first-line imaging modality, especially for the early detection of muscle changes in rheumatic diseases. The sensitivity to change of muscle ultrasound, particularly its responsiveness to interventions such as drug treatments, supplements, and regular physical exercise, is an important area that requires further investigation. Additionally, combining ultrasound with other modalities, such as DXA or functional performance tests, may offer a more comprehensive understanding of sarcopenia, ultimately improving early detection and intervention strategies.

Looking ahead, exploring automated image analysis through AI and machine learning could enhance the accuracy and efficiency of ultrasound assessments of sarcopenia [116]. Specifically, the evaluation of muscle echogenicity and quantitative methods, such as histographic analysis, can be applied to ultrasound images using specialized software to analyze pixel intensity (i.e. image echogenicity). Indeed, the opportunity to consider implementation of a digital measurement into the ultrasound machine is a fascinating hypothesis. Finally, the role of SWE in patients with sarcopenia has been scarcely explored, particularly in those with rheumatic diseases [112, 117].

## Nerves

Carpal tunnel syndrome (CTS) is the most common neurological manifestation of rheumatic diseases, especially RA [118]. While nerve conduction studies are traditionally used to confirm the diagnosis of CTS, ultrasound has emerged as a powerful tool for assessing median nerve and carpal tunnel pathology [119].

**Fig. 5:**
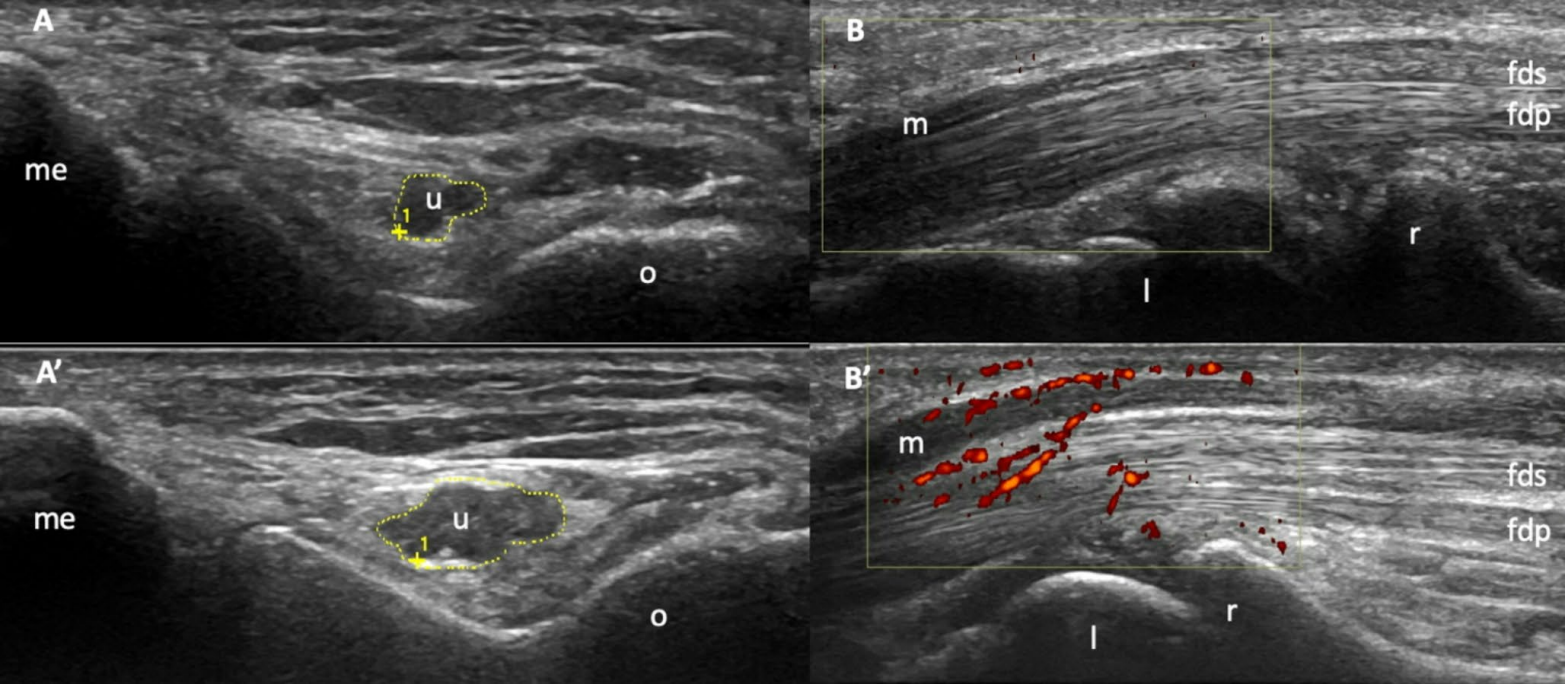
Ultrasound in the assessment of nerves (compressive neuropathty). **A** shows a transverse view of a normal ulnar nerve at the elbow in a healthy subject, while **A’** displays an enlarged ulnar nerve (increased cross-sectional area) in a patient with cubital tunnel syndrome. **B** presents a longitudinal view of a normal median nerve at the wrist in a healthy subject, and **B’** shows an enlarged median nerve with power Doppler signal (red spots) in a patient with carpal tunnel syndrome. *fds* = flexor digitorum superficialis; *fdp* = flexor digitorum profundus; *l* = lunate; *m* = median nerve; *me* = medial epicondyle; *o* = olecranon; *r* = radius; *u* = ulnar nerve

In CTS, the key ultrasound feature is an increased cross-sectional area (CSA) of the median nerve at the sites of compression, which has been shown to be both sensitive and specific for diagnosis and severity assessment [120]. CSA thresholds have been proposed (generally, a CSA above 12 mm² is considered pathological), but these can vary according to anthropometric parameters [121]. The detection of Doppler signal in the median nerve can further enhance diagnostic specificity [122]. Pictorial examples of nerve pathology on ultrasound are illustrated in Fig. 5. Ultrasound also helps detect structural changes in the tissues adjacent to the nerve that may cause compression, such as synovitis, tenosynovitis, and crystal deposits [123]. Finally, ultrasound guidance improves the precision of corticosteroid injections, reducing the odds of CTS recurrence within one year by 55% compared to blind injections [124].

In patients with RA, CTS may exhibit distinct ultrasound characteristics compared to idiopathic CTS. The most common findings in RA-related CTS are inflammation-related changes affecting the structures surrounding the median nerve, such as tenosynovitis of the finger flexor tendons and synovitis at the radiocarpal joint [125]. In contrast, idiopathic CTS is primarily characterized by an increased CSA of the median nerve, with or without an intra-neural Doppler signal. Other compressive neuropathies assessable via ultrasound include cubital tunnel syndrome, Guyon’s syndrome, radial tunnel syndrome, tarsal tunnel syndrome, and others [119].

The main limitation of ultrasound in the evaluation of nerve pathology is its limited ability to assess deeper nerves, especially those located in areas covered by bone or complex anatomical structures. In addition, while ultrasound can visualize morpho structural nerve changes (i.e. nerve size, structure, entrapment or compression), it does not provide direct information about nerve conductivity or the functional status of the nerve.

Emerging evidence supports the role of AI in assessing nerve involvement, showing excellent agreement between the algorithm and sonographers in measuring CSA of the median nerve [126]. Additionally, some studies have examined the value of SWE in measuring stiffness of the median nerve, demonstrating high diagnostic performance for CTS, especially if combined with CSA (specificity 100% and sensitivity 93%) [127].

## Skin and Subcutaneous Tissue

### Scleroderma

Scleroderma, or skin thickening, is a hallmark feature of SSc and is particularly relevant for diagnosing and monitoring disease progression [128]. Historically, the Modified Rodnan Skin Score (mRSS), a palpation-based scoring method, has been used to assess skin thickening in both clinical practice and trials [129]. While mRSS is simple and quick, it has shown variable inter-observer reliability, limited sensitivity to small changes, and an inability to differentiate between the various pathological phases of the skin (edematous, fibrotic, and atrophic) [130]. Consequently, there is growing interest in alternative methods, particularly ultrasound, for assessing skin involvement in SSc.

**Fig. 6:**
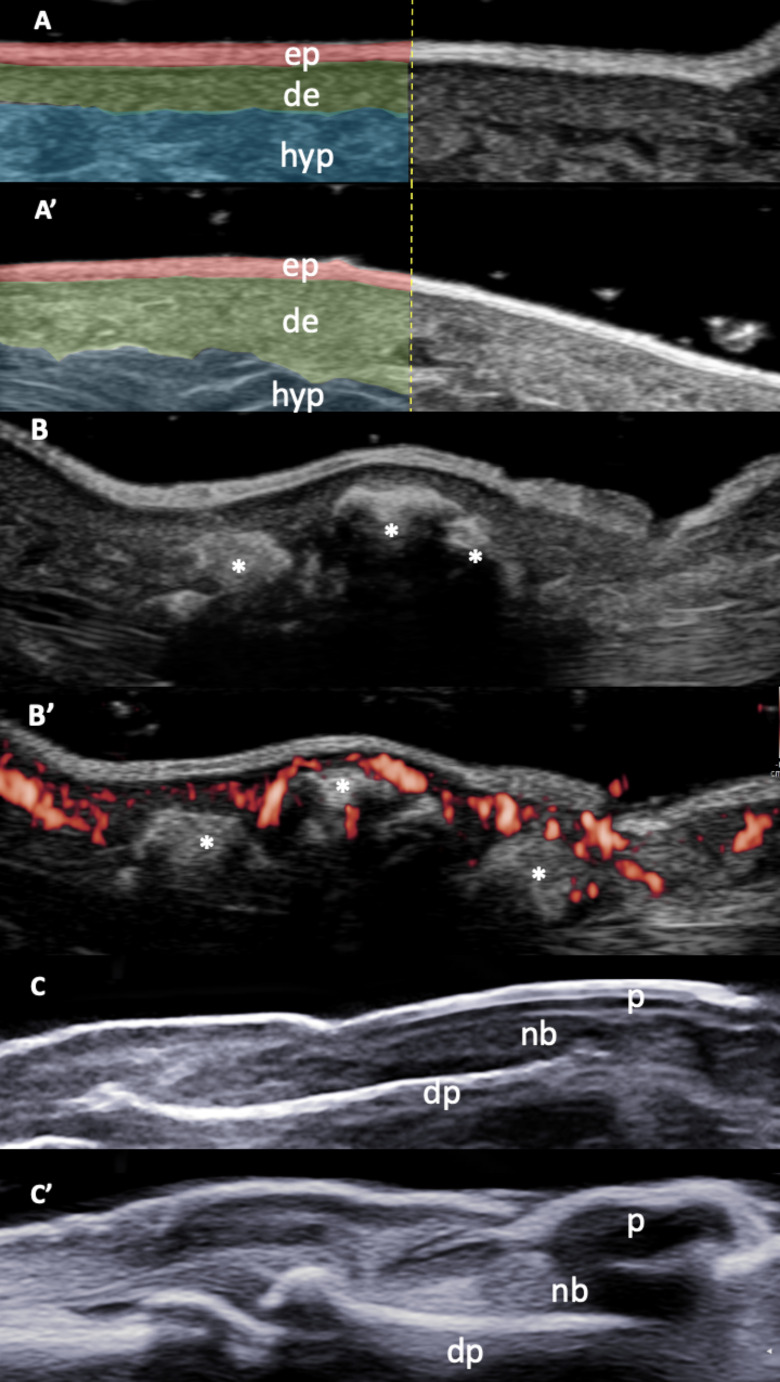
Ultrasound in the assessment of skin (scleroderma), sub-cutaneous soft tissues (calcinosis cutis) and nails (nail psoriasis). **A** presents a comparative ultrasound image of skin layers in the atrophic phase of systemic sclerosis, obtained with a high-frequency probe (22 MHz). The epidermis (red) appears as a thin hyperechoic layer, while the dermis (green) is hypoechoic with tightly packed, regular connective tissue fibers. The subcutaneous tissue (blue) is more hypoechoic, with looser connective tissue fibers. In **A’**, the epidermal, dermal, and subcutaneous layers of a healthy subject are clearly delineated, following the same echogenic patterns as in **A**, though with notable differences in thickness, especially in the dermis. **B** shows an ultrasound image of subcutaneous calcinosis, highlighting calcifications of variable shapes and sizes (asterisks) accompanied by acoustic shadowing. In **B’**, a surrounding power Doppler signal (red spots) is visible in a patient with systemic sclerosis. Finally, **C** provides an ultrasound comparison of a physiological nail, showing a normal trilaminar structure, while **C’** depicts a psoriatic nail with loss of the ventral plate and increased nail bed thickness. *de* = dermis; *dp* = distal phalanx; *ep* = epidermis; *hyp* = hypodermis; *nb* = nail bed; *p* = nail plate. **A**, **A’**, **B**, and **B’** kind courtesy of Prof. L. Idolazzi

With the advent of ultra-high-frequency probes (50–70 MHz), it is now possible to assess even minimal changes in superficial structures, such as the epidermis, dermis, and hypodermis, with remarkable detail (Fig. 6). Some studies suggest that in patients with SSc and puffy fingers, the hypodermis is affected first, showing thickening, followed by dermal thickening in the fibrotic phase. In the atrophic phase, both the dermis and hypodermis become reduced [131]. Thus, the ability of ultrasound to detect changes in these distinct skin layers provides valuable insights into the pathogenesis of skin involvement in SSc. Additionally, ultrasound has identified abnormalities in SSc patients with a normal mRSS, suggesting its potential to detect subclinical skin involvement [132].

Significant work remains before skin ultrasound becomes routine for patients with SSc, and to fulfil the OMERACT filter criteria of truth, discrimination, and feasibility [133]. The World Scleroderma Foundation has initiated efforts to standardize the technique [134]. A recent study proposed cut-off values for skin thickness in healthy subjects, with further research needed to validate these cut-offs in patients with SSc [135]. Future directions also include correlating ultrasound findings with molecular patterns identifiable in biopsies, as well as with the evolving phases of skin involvement [136, 137].

Recent studies have examined the role of SWE, showing that skin stiffness measured with SWE is significantly higher in SSc patients than in healthy individuals. SWE has demonstrated greater reliability than mRSS, suggesting it may aid in the diagnosis and monitoring of skin involvement in SSc [138, 139].

### Calcinosis Cutis

Calcinosis cutis refers to the presence of calcium deposits in the skin and subcutaneous tissues. This rare manifestation can be found in dermatomyositis (especially the juvenile form), SSc, MCTD, and rarely, SLE [140]. On ultrasound, these deposits typically appear as well-defined hyperechoic areas with or without posterior acoustic shadowing [141, 142] and/or surrounding Doppler signal (Fig. 6). Ultrasound has demonstrated a sensitivity of 89% for detecting calcinosis in patients with SSc when compared to conventional radiography [143].

Questions about the diagnostic accuracy, inter-operator variability, and standardization of ultrasound for assessing calcinosis cutis remain unresolved. Subcutaneous calcifications may also arise in other conditions, such as chronic renal failure, hydroxyapatite deposition disease, or vascular calcification, which can mimic CTD-related calcinosis cutis. Furthermore, the role of ultrasound in monitoring changes in calcinosis over time is uncertain, as existing studies have included only a limited number of patients [144]. Finally, the clinical significance of the Doppler signal, such as its association with pain or potential reparative mechanisms, also remains to be fully clarified.

### Psoriatic Plaque and Nail Psoriasis

Psoriasis is a chronic inflammatory disorder that leads to the formation of lesions on the skin (i.e. psoriatic plaques) due to rapid skin cell turnover caused by a dysregulated immune response [145]. Using high-frequency probes, psoriasis plaques exhibit various ultrasound morpho-structural alterations affecting both the epidermis and dermis [146]. The best described ultrasound sign for plaque psoriasis is a thickening of the epidermis and dermis compared to the healthy skin [147]. In particular, this lesion presents a “four-band layout”, consisting of a hyperechoic epidermal band, a hypoechoic band (dermal papillae), a second hyperechoic band (reticular dermis), and a final hypoechoic subcutaneous layer. These hypoechoic bands can lead to acoustic shadowing, and an increase in Doppler signal within the hypoechoic band in the upper dermis has also been noted [148].

Nail psoriasis (NP) is a significant manifestation of psoriasis [149]. The nail complex, comprising the matrix, nail bed, hyponychium, and eponychium, can exhibit various changes. In NP, alterations in the nail matrix may lead to leukonychia, pitting, and crumbling, while nail bed changes can manifest as onycholysis and subungual hyperkeratosis [149]. High-frequency linear probes (22–24 MHz) are recommended for optimal imaging of NP [150]. Dermatologists and rheumatologists are increasingly interested in the relationship between nail involvement and PsA, particularly the anatomical and functional link between entheses, nails, and the distal interphalangeal joints [151]. Ultrasound could be especially beneficial in exploring this connection, as it allows for the simultaneous examination of these structures. Several ultrasound classifications for NP have been proposed [152, 153]. The loss of ventral plate integrity (i.e. the loss of the typical trilaminar structure) is characteristic of PsA patients (Fig. 6).

In conclusion, with the growing interest in targeted therapies for psoriasis and PsA, ultrasound may provide valuable insights into the progression of both skin and NP. However, to date, only a few small observational studies have evaluated its use in monitoring treatment responses, such as to apremilast and methotrexate [154, 155]. Currently, there is no consensus on the most effective scoring method for clinical or research use, and further validation is needed. Challenges such as interindividual variability in skin and nail changes, as well as the need for high-frequency probes, continue to hinder its widespread adoption in clinical practice, particularly in rheumatology.

## Novel Potential Targets for Ultrasound Extra-Articular Assessment in Rheumatic Diseases

Recent studies have suggested novel potential uses of ultrasound for assessing extra-articular manifestations in patients with rheumatic diseases.

For example, in Behçet’s syndrome, ultrasound enables the detection of active inflammation in venous vessels, such as the femoral vein, and the identification of complications such as stenosis, occlusions, aneurysms, and thrombosis, offering non-invasive diagnostic possibilities [156].

In GCA, there is growing interest in using ultrasound to evaluate ocular vessels, which may have prognostic implications. A recent study demonstrated that patients with visual symptoms show reduced retinal artery flow and increased optic nerve diameter compared to controls, suggesting that these signs could serve as markers of disease progression [157].

Ultrasound has also been explored in IgG4-related retroperitoneal fibrosis, where it can reveal a well-defined hypoechoic or anechoic mass with irregular contours, sometimes accompanied by hydronephrosis or hydroureter. However, it is important to note that ultrasound has limited sensitivity compared to CT, detecting the disease in only 25% of cases [158].

In SSc, intestinal vasculopathy is a key pathogenic factor in gastrointestinal involvement. Consequently, ultrasound has been used to examine the mesenteric arteries in these patients, revealing a reduction in both vessel caliber and reverse velocity compared to controls [159]. Other studies have focused on vascularization in SSc patients with Raynaud’s phenomenon, analyzing blood flow at the nail fold and fingertip, and ulnar artery [160, 161]. Both studies found that mean vascular intensities were lower in affected patients compared to healthy individuals.

Another promising application concerns the lacrimal glands in SjD. Although no established ultrasound scoring systems currently exist to evaluate glandular alterations, pathological findings (e.g. changes in gland size, parenchymal homogeneity, and the presence of hypoechoic areas) have been documented, suggesting a potential role for ultrasound (especially very high frequency probes) in the assessment of these structures [162].

## Conclusions

Ultrasound is playing an increasingly important role in assessing extra-articular manifestations across a range of rheumatic diseases. LUS is proving especially valuable in diagnosing and managing ILD, a key feature of CTDs, particularly SSc. In LVV, such as GCA and TAK, ultrasound has transformed early diagnosis; notably, the halo sign on the temporal artery is now included in the latest ACR/EULAR classification criteria.

Ultrasound is also effective for detecting salivary gland changes in SjD, potentially facilitating diagnosis and reducing the need for invasive biopsies. Furthermore, it aids in assessing muscle disorders, including IIM and sarcopenia, as well as in diagnosing peripheral neuropathies like CTS. It is also valuable for evaluating skin and subcutaneous involvement in patients with SSc, with growing interest in using ultrasound to assess skin and nail involvement in psoriasis. Additionally, emerging areas for extra-articular assessment have been described in several rheumatic diseases.

Despite these benefits, the application of ultrasound in this context faces certain challenges. Key limitations include a lack of standardized ultrasound protocols and universally accepted scoring systems, variability among operators, and the need for specialized training and equipment.

New techniques, such as SWE, the use of high-frequency probes, and the integration of AI, hold promise for improving the accuracy and clinical utility of ultrasound in evaluating extra-articular manifestations in rheumatic diseases.

## Data Availability

No datasets were generated or analysed during the current study.
